# Factors which influence the length of an out-of-hours telephone consultation in primary care: a retrospective database study

**DOI:** 10.1186/1472-6963-12-430

**Published:** 2012-11-26

**Authors:** Mohammed A Mohammed, Gill Clements, Elaine Edwards, Helen Lester

**Affiliations:** 1 Primary Care Clinical Sciences, University of Birmingham, Birmingham, England, University of Birmingham, Edgbaston, Birmingham, B15 2TT, UK; 2 Shropshire Doctors Co-operative Limited, Shrewsbury, UK

**Keywords:** Telephone consultation, Duration, Efficiency, Nurse practitioner, General practitioner, Out-of-hours, After-hours, Emergency care, Primary care, Telephone, Triage

## Abstract

**Background:**

Given the increasing use of telephone consultation it is important to determine the factors which influence the length of a telephone consultation.

**Method:**

Analysis of 128717 telephone consultations during January to December 2011 to a National Health Service (NHS) out-of-hours primary care service provider in Shropshire and Telford and Powys, England, involving 102 General Practitioners (GPs) and 36 Nurse Practitioners (NPs). Telephone consultation conclude with one of three outcomes – advice only, the patient is invited to a face-to-face consultation with a GP or NP at a nearby health centre (known as a base visit) or the patient is visited at home by a GP or NP (known as home visit). Call length was analysed by these outcomes.

**Results:**

The overall mean call length was 7.78 minutes (standard deviation (SD) 4.77). Calls for advice only were longest (mean 8.11 minutes, SD 5.17), followed by calls which concluded with a base visit (mean 7.36 minutes, SD 4.08) or a home visit (mean 7.16 minutes, SD 4.53). Two primary factors influenced call length. Calls by GPs were shorter (mean 7.15 minutes, SD 4.41) than those by NPs (mean 8.74 minutes, SD 5.31) and calls designated as a mental health call were longer (mean 11.16 minutes, SD 4.75) than all other calls (mean 7.73 minutes, SD 7.7).

**Conclusions:**

Telephone consultation length in the out-of-hours setting is influenced primarily by whether the clinician is a GP or a NP and whether the call is designated as a mental health call or not. These findings suggest that appropriate attempts to reduce the length of the telephone consultations should focus on these two areas, although the longer consultation length associated with NPs is offset to some extent by their lower employment costs compared to GPs. Nonetheless the extent to which the length of a telephone consultation impacts on subsequent use of the health service and correlates with quality and safety remains unclear.

## Background

Telephone consultations are an increasingly common [1-4], feature of modern healthcare, especially in the primary care setting. Over the period 1995 to 2006, the proportion of all General Practitioner (GP) consultations conducted on the telephone trebled whilst the proportion recorded as home visits halved [5]. Telephone consultations appear to be acceptable to patients [6] and also offer time, efficiency and cost-saving benefits but questions about safety, and cost still remain [7-9]. Nevertheless, perhaps driven primarily by the need to reduce costs, there is an ongoing commitment in the NHS to improving access to primary care by extending opening hours and expanding the provision of telephone consultations in primary care [10]. A key driver of cost and hence as a candidate performance indicator [11,12], in the face-to-face consultation is the length of the consultation; but whilst studies internationally have provided insight into factors which influence the length of face-to-face consultations in primary care [13-17], much less is known about what factors influence the length of telephone consultations in primary care. The aim of this study, using telephone consultations data collected from an out-of-hours primary care service provider (known as ShropDoc [18]) over a twelve month period, was to determine which factors influenced the duration of the telephone consultation.

## Methods

### Data source

ShropDoc is a not-for-profit GP Cooperative Company that provides urgent medical services for patients when their primary care GP surgery is closed and whose needs cannot safely wait until the surgery is next open. Typically the out-of-hours times are 1800h to 0800h Monday to Friday and all hours for weekends and bank holidays. Shropdoc provides services to 600,000 patients in Shropshire, Telford and Wrekin and Powys and handles approximately 140,000 calls and over 50,000 face-face patient contacts per year [16]. Contact between patient and clinician commences with a telephone call. A call handler takes the patients details, identifies immediate life threatening conditions using a “trigger list” (diverts these to the 999 ambulance service) and then prioritises the call (i.e. urgent or routine). The patient is then called back by a GP or a Nurse Practitioner (NP) and this call is defined as the telephone consultation. This concludes with one of three outcomes – advice only, the patient is invited to a face-to-face consultation with a GP or NP at a nearby health centre (known as a base visit) or the patient is visited at home by a GP or NP (known as a home visit). Neither GPs nor NPs use computer assisted algorithms/decision support systems in ShropDoc. Upon receipt, the telephone calls go into a queue – the urgent calls are returned within 20 minutes and the routine with 60 minutes. The start of a consultation occurs when the clinician opens the patient record and makes the call and ends when the clinical record is closed by that clinician and so this includes time to read existing notes and write additional notes. We analysed all of ShropDocs out-of-hours telephone consultations in the twelve month period (January to December 2011). In general the majority of calls are handled on a first-in-first-out basis but this sequence can be overridden by the clinician, especially where there is an urgent clinical need. The work was undertaken as part of the regular audit and service evaluation undertaken by ShropDOC.

### Statistical analysis

Call length is reported in minutes and summarised as means and standard deviation (SD) as well as medians and inter-quartile ranges (IQR), although for statistical modelling we used means. Our primary analysis involved Classification and Regression Trees (CART) which are a statistical data mining based technique for constructing trees by recursively splitting or partitioning patients into homogenous groups [19] and have been used to support medical decision making [20-22], although their use is still somewhat novel. Tree models can reflect human decision making and are intuitive to interpret because they have a simple visual presentation which starts by identifying the most important predictor variables, naturally incorporates interaction effects and identifies cut-offs for continuous covariates. As first developed, CART, could lead to quite large tree models, but recent work has incorporated p-value based tree modelling, known as conditional classification trees, which yield smaller tree models whilst simultaneously controlling for multiple testing and are available in the *Party* Package [23] in R [24].

Our purpose in using the conditional tree models is to uncover the factors which influence the length of the telephone consultation as opposed to predictive modelling [20]. The factors that we considered were based on variables that were routinely collected in ShropDoc's database, of which we selected the following: patient's age, patient's gender, date/time call started, date/time call ended, outcome of the call (advice, base visit or home visit) and whether the clinician was a GP or a NP. ShropDoc also classify their calls into broad clinical headings using their internally developed categories. The majority of calls were classified as "Other" (73.6%), but we did flag calls which were classified as, Respiratory n=11795, Pharmacy/Medication n=6610, Minor Injury n=4807, Mental Health n=2666, Acute Retention and/or Catheter n=1806, Palliative Care n=2336, Deceased n=1023 and Dental calls n=281 in the tree models. We also included variables for identifying calls that were made on a Saturday or a Sunday because experience from ShropDoc indicated that these days may be different in terms of workload and healthcare service provision.

We produced conditional trees for each outcome – advice, base visit and home visit. Each node in the tree shows the factor (with its statistical significance, which we predefined at p<0.01) and the end of the branches are rectangles which show the sample size (n) and the mean call duration (y) in minutes. Each node in the tree also has a node identification number for ease of reference.

## Results

**Figure 1:**
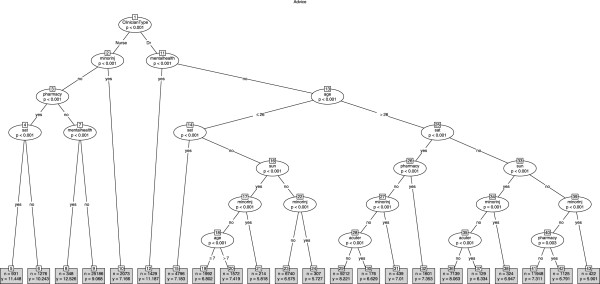
Tree model identifying factors associated with call duration for all calls that ended with advice only.

**Figure 2:**
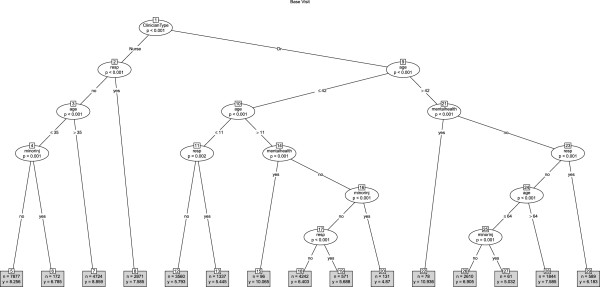
Tree model identifying factors associated with call duration for all calls that ended in a base visit.

**Figure 3:**
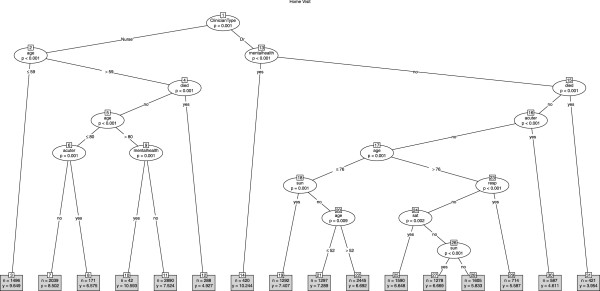
Tree model identifying factors associated with call duration for all calls that ended in a home visit.

In the twelve month period (January to December 2011) there were 128717 telephone consultations involving 102 doctors and 36 NPs. The mean age of patients was 42.15 years (SD 30.25) of whom 57.5% were female (38634/ 128717). The mean telephone consultation length was 7.78 minutes (SD 4.77). Almost two-thirds of the calls ended with advice only (61.7%, 79379/67245), about a quarter ended with a base visit (24.0%, 30763/128717) and 14.4% ended with a home visit (18575/128717). We produced conditional tree models for each call outcome (see Figures 1, 2, 3). Calls designated as a mental health call were longer (mean 11.16 minutes, SD 4.75) than all other calls (mean 7.73 minutes, SD 7.7). 

### Advice only

The mean call length for calls that ended with advice only was 8.11 minutes (SD 5.17). The tree model (Figure 1) showed that the primary predictor (node 1) of call length was whether the call involved a GP (mean 7.52 minutes, SD 4.68) or a NP (mean 9.10 minutes, SD 5.77), although GPs dealt with older patients (GP mean patient age 45.73 years, SD 29.68 vs NP mean patient age 35.40 years, SD 29.04). For NPs and GPs, three types of calls featured in the tree model (mental health, minor injuries and pharmacy related) whilst the patients age and calls designated as acute retention and/or catheter related featured only in the GPs half of the tree model. Calls designated as mental health calls were the longest durations for GPs (node 11: mean 11.19 minutes, SD 7.68) and NPs (node 7: mean 12.53 minutes, SD 9.42), whereas calls designated as minor injuries were the shortest duration for GPs (nodes 21,24,31 and 37) and NPs (node 10). Call length was also influenced by Saturday and/or Sunday - for example, for GPs and NPs, pharmacy related calls were significantly longer on a Saturday compared to other days of the week. The patient's gender did not feature in the tree model.

### Base visit

The overall mean call length for calls that ended with a request for the patient to be booked into a base appointment for a face-to-face consultation with a GP or a NP was 7.36 minutes (SD 4.08). The tree model (Figure 2) again showed that the primary predictor (node 1) of call length was whether the call was involved a GP (GP mean 6.40 minutes, SD 3.53) or a NP (mean 8.30 minutes, SD 4.35) although GPs dealt with older patients (GP mean age 31.15 years ; NPs mean age 27.94 years). For GPs, mental health calls were the longest in length (node 22, node 15) although NPs took longer with mental health calls than GPs (GPs mean 10.46 minutes, n=174 ; NPs mean 11.74 minutes n=102) the mental health calls were absent from the tree model. For both GPs and NPs, minor injury calls were the shortest (nodes 6, 20 and 27), although NPs took longer than GPs. Interestingly, there were no apparent Saturday/Sunday association in the tree model. Once again, the patient's gender did not feature in the tree model and perhaps unsurprisingly neither did pharmacy related calls, minor injury calls or calls involving the notification of a death.

### Home visit

The overall mean call length for calls that ended with a home visit was 7.16 minutes (SD 4.53). The tree model (Figure 3) again showed that the primary (node 1) predictor of call length was whether the call was made by a GP (mean 6.57 minutes, SD 4.07) or a NP (mean 8.16 minutes, SD 5.07) although this time GPs and NPs dealt with patients of a similar age profile (GPs mean age 71.03 years; NPs mean age 71.72 years). Mental health calls were the longest in length with little difference between GPs and NPs (GPs mean 10.24 minutes, NPs mean 10.59 minutes). Calls where the caller notified ShropDoc of a death were the shortest in duration, with GPs taking an average of 3.95 minutes (node 31) whilst NPs took an average of 4.93 minutes (node 12). Some influence of Saturday and/or Sunday was seen in the GP section of the tree model but not in the NP sections. In addition, the GP section of the tree identified respiratory disease as a factor (node 23) associated with shorter calls, whilst the NPs section of the tree identified acute retention and/or catheter related (node 8) as being associated with shorter duration.

### GPs and NPs

Since the tree models consistently identified the clinicians profession (GP or NP) as a key factor in determining telephone consultation call lengths we undertook additional analyses comparing GPs and NPs (see Table 1), which showed some systematic differences. GPs were more likely to conclude a call with advice only (GPs 64.93% vs NPs 56.91%), whilst NPs were less likely to conclude a call with a home visit (GPs 15.26% vs NPs 13.22%) but more likely to conclude a call with a base visit (GPs 19.81% vs NPs 29.86%).

**Table 1:** Some characteristics of telephone consultations by GPs and NPs

**Call Outcome**	**Characteristic**	**GP**	**NP**
All	Number of calls	76333	52384
	Mean Duration (mins)	7.15	8.74
	SD Duration	4.41	5.31
	Median Duration (mins)	6.07	7.93
	IQR	5	6
	Mean Age (years) (SD)	46.47 (30.17)	37.97 (30.05)
	Female(%)	43513 (57.00)	30569 (58.36)
Advice Only	Number of calls	49565 (64.93)	29814 (56.91)
	Mean Duration (mins)	7.52	9.10
	SD Duration	4.68	5.77
	Median Duration (mins)	6.78	8.0
	IQR	5.12	7
	Mean Age (years) (SD)	45.37 (29.68)	35.40 (29.04)
	Female(%)	28704 (57.91)	17648 (59.92)
Base Visit	Number of calls	15119 (19.81)	15644 (29.86)
	Mean Duration (mins)	6.40	8.30
	SD Duration	3.53	4.35
	Median Duration (mins)	5.88	7.33
	IQR	4	4.78
	Mean Age (years) (SD)	31.15 (26.03)	27.94 (24.31)
	Female(%)	8362 (55.31)	8965 (57.31)
Home Visit	Number of calls	11649 (15.26)	6926 (13.22)
	Mean Duration (mins)	6.57	8.16
	SD Duration	4.07	5.07
	Median Duration (mins)	6	7
	IQR	4.2	5.58
	Mean Age (years) (SD)	71.03 (20.62)	71.72 (22.62)
	Female(%)	6447 (55.34)	3956 (57.12)

IQR is the inter-quartile range. SD is standard deviation.

## Discussion

### Summary of main findings

Using a large volume of telephone consultations over a one year period in the out-of-hours setting in primary care we were able to estimate that the mean call length was 7.78 minutes (SD 4.77). The fact that GPs and NPs have persistently different call lengths, even after controlling for some patient characteristics, and differing rates of advice and base visits suggests that there may be some systematic differences in the communication and decision making process of GPs and NPs. Mental health calls took the longest, for GPs and NPs, irrespective of the call outcome (advice, base visit and home visit). Whilst studies have reported longer face-to-face consultations for patients with mental health related issues in primary care [13,14,25] this is the first time that this has been reported in the telephone consultation context. We also found mental health calls with NPs were longer than those with GPs, although this difference was most pronounced for calls ending in advice and became less pronounced for base and home visits.

### Comparison with existing literature

The mean call duration of 7.78 minutes is, perhaps unsurprisingly, shorter than the average consultation length seen in English general practice (9.4 minutes), but is not dissimilar to face-to-face consultations reported [7] for Germany (7.6 minutes SD 4.3) and Spain 7.8 minutes (SD 4.0). However, as Deveugele et al [7] noted, determining the length of face-to-face consultation is not straightforward (e.g. because of different operational definitions for the start and end times adopted by different researchers). This applies equally to telephone consultations because in our study the times included reading/writing of notes on the computer system. Furthermore, the extent to which meaningful comparisons between countries can be made in respect of consultation length, be it face-to-face or telephone based consultations, is unclear because of differences in health systems and the role of the GPs [7-9]. Whilst telephone based out-of-hours service provision is seen in several countries, there are differences, with varying levels of clinical qualified staff (including lay people) in the front-line, working with/without protocols and with/without second level triage from experienced GPs, making it difficult to meaningfully compare call durations internationally. Even in the English NHS, there are several models of out-of-hours call centres.

### Strengths and limitations

Our study provides a valid comparison between GPs and NPs in the out-of-hours setting but the findings are not generalisable to settings where decision support systems are routinely used or where NPs are not working alongside GPs. From an employment cost perspective, the longer telephone consultations lengths associated with NPs are offset, to some extent, by their lower employment costs. This also needs to be balanced against possibly reduced access for other patients and the lower advice rates and higher rates of base visits delivered by NPs. Nevertheless the extent to which the length of a telephone consultation impacts on subsequent use of the health service and correlate with quality and safety remain unclear.

Our study did not set out to test any specific hypotheses although we have generated several for possible future testing. We did not assess the quality or safety of the telephone consultation, analyse the reason or urgency for the call, the appropriateness of the clinical outcomes, including patient satisfaction or analyse the variation within GPs and NPs. These issues merit further study.

### Implications for clinical practice

Health systems need to continually find new ways to provide safe, cost effective care. Primarily because of cost savings and supported by evidence from RCTs that NPs are generally regarded as safe in telephone consultations (although some concern was noted in a Dutch study [26,27],), the deployment of NPs in roles more traditionally associated with doctors is increasing [28]. ShropDoc have set up their service so that patients may consult a GP or a NP, irrespective of the nature of the call. Indeed this novel model of service provision, where GPs and NPs work alongside each other, may offer other benefits in building teamwork and shared expertise, despite longstanding differences of power, pay, status and gender between these two healthcare professionals [18].

## Conclusions

Telephone consultation length in the out-of-hours setting is influenced primarily by whether the clinician is a GP or a NP and whether the call is designated as a mental health call or not. The result suggests that appropriate attempts to reduce the length of the telephone consultations should focus on these two areas, although the longer consultation length associated with NPs is offset to some extent by their lower employment costs compared to GPs. Nevertheless the extent to which the length of a telephone consultation impacts on subsequent use of the health service and correlates with quality and safety remains unclear.

## Competing interests

GC & ED are employees of ShropDOC. MAM has undertaken consultancy work for ShropDOC. HL has no conflicts of interest. The authors declare that they have no competing interests.

## Authors’ contributions

MAM conceived of the study with GC and ED and undertook the analysis and drafted the manuscript. HL assisted with interpretation and manuscript writing. All authors contributed to the manuscript and have all read and approved the final manuscript.

## Pre-publication history

The pre-publication history for this paper can be accessed here:

http://www.biomedcentral.com/1472-6963/12/430/prepub

## References

[B1] Toon PD Using telephones in primary care BMJ 2002 324 7348 1230 1231 10.1136/bmj.324.7348.1230 12028963 PMC1123203

[B2] Innes M Skelton J Greenfield S A profile of communication in primary care physician telephone consultations: application of the Roter Interaction Analysis System Br J Gen Pract 2006 56 526 363 368 16638252 PMC1837845

[B3] Males T Telephone consultations in primary care: a practical guide 2007 RCGP, Springer London Ltd

[B4] Burton C Telephone Consultations 2009 uk: Patient.co May http://www.patient.co.uk/doctor/Telephone-Consultations.htm#ref20

[B5] Hippisley-Cox J Fenty J Heaps M Trends in consultation rates in general practice 1995 to 2006: Analysis of the QRESEARCH database 2007 NHS Information centre http://www.ic.nhs.uk/webfiles/publications/gp/QRESEARCH%20Consultation%20Rates%20Report%20FINAL.pdf

[B6] Car J Sheikh A Telephone consultations BMJ 2003 326 966 969 10.1136/bmj.326.7396.966 12727771 PMC153854

[B7] Bunn F Byrne G Kendall S Cochrane.org Telephone consultation and triage: effects on health care use and patient satisfaction Cochrane Database Syst Rev 2004 4 CD004180 pub2. BMJ (337):645 15495083 10.1002/14651858.CD004180.pub2

[B8] Giesen P Smits M Huibers L Grol R Wensing M Quality of after-hours primary care: a narrative review of the Dutch solution Ann Intern Med 2011 155 108 113 21768584 10.7326/0003-4819-155-2-201107190-00006

[B9] Huibers L Smits M Renaud V Giesen P Wensing M Safety of telephone triage in out-of-hours care: A systematic review Scand J Prim Health Care 2011 29 4 198 209 10.3109/02813432.2011.629150 22126218 PMC3308461

[B10] Carson D Clay H Stern R Blackledge C Cowper A New ideas and resources for clinical commissioners on the journey towards integrated 24/7 urgent care 2011 Primary Care Foundation http://www.primarycarefoundation.co.uk/files/PrimaryCareFoundation/Downloading_Reports/Reports_and_Articles/Urgent_Care_Commissioning/Breaking%20the%20Mould%20RELEASE.pdf

[B11] Wilson A Childs S The relationship between consultation length, process and outcomes in general practice: a systematic review Br J Gen Pract 2002 52 1012 1020 12528590 PMC1314474

[B12] Carr-Hill R Jenkins-Clarke S Dixon P Pringle M Do minutes count? Consultation lengths in general practice J Health Serv Res Policy 1998 3 4 207 213 10187199 10.1177/135581969800300405

[B13] Britt HC Valenti L Miller GC Determinations of consultation length in Australian general practice MJA 2005 183 68 71 16022609 10.5694/j.1326-5377.2005.tb06924.x

[B14] Raynes NV Cairns V Factors contributing to the length of general practice consultations J R Coll Gen Pract 1980 30 496 498 7452585 PMC2159660

[B15] Wescott R The length of consultation in general practice J R Coll Gen Pract 1983 30 496 498 PMC2159660 7452585

[B16] Wilson A Consultation length in general practice: a review BJGP 1991 41 1 19 22 2031756 PMC1371626

[B17] Deveugele M Derese A Brink-Muinen A Bensing J Maeseneer JD Consultation length in general practice: cross sectional study in six European countries BMJ 2002 325 472 477 10.1136/bmj.325.7362.472 12202329 PMC119444

[B18] ShropDOC About ShropDOC 2012 http://www.shropdoc.org.uk/about.php, accessed Mar

[B19] Breiman L Friedman JH Olshen RA Stone CJ Classification and regression trees 1984 Monterey, CA: Wadsworth & Brooks/Cole Advanced Books & Software

[B20] Steyerberg EW Clinical Prediction Models. A practical approach to development, validation and updating 2009 Springer

[B21] Harper PR A review and comparison of classification algorithms for medical decision making Health Policy 2005 71 315 331 10.1016/j.healthpol.2004.05.002 15694499

[B22] Podgorelec V Kokol P Stiglic B Rozman I Decision Trees: An Overview and Their Use in Medicine J Med Syst 2002 26 5 10.1023/a:1016409317640 12182209

[B23] Hothorn T Hornik K Zeileis A Unbiased Recursive Partitioning: A Conditional Inference Framework J Comput Graph Stat 2006 15 3 651 674 10.1198/106186006X133933

[B24] R Development Core Team R: A language and environment for statistical computing 2011 Vienna, Austria: R Foundation for Statistical Computing URL http://www.R-project.org

[B25] Hutton C Gunn J Do longer consultations improve the management of psychological problems in general practice? A systematic literature review BMC Health Serv Res 2007 7 71 10.1186/1472-6963-7-71 17506904 PMC1890290

[B26] Giesen P Ferwerda R Tijssen R Mokkink H Drijver R Van den Bosch W Safety of telephone triage in general practitioner cooperatives: do triage nurses correctly estimate urgency? Qual Saf Health Care 2007 16 181 184 10.1136/qshc.2006.018846 17545343 PMC2465002

[B27] Car J Koshy E Bell D Sheikh A Telephone triage in out of hours call centres. Concerns about quality and safety highlight the need for further evaluation BMJ 2008 337 a1167 10.1136/bmj.a1167 18790813

[B28] Salvage J Smith R Doctors and NPs: doing it differently BMJ 2000 320 1019 1020 10.1136/bmj.320.7241.1019 10764342 PMC1117926

